# Perspektiven auf das Lebensende: eine systematische Erhebung bei Patienten mit amyotropher Lateralsklerose

**DOI:** 10.1007/s00115-024-01769-4

**Published:** 2024-11-15

**Authors:** Clemens Eickhoff, Bettina Schöne-Seifert, Dagmar Kettemann, Eike Bormann, Torsten Grehl, Matthias Boentert, Jan C. Koch, Carolina Schmitt, Bertold Schrank, Carsten Schröter, Thomas Meyer

**Affiliations:** 1 Klinik für Neurologie und Klinische Neurophysiologie, Helios Klinik und MVZ Kassel, Bergmannstr. 30, 34121 Kassel, Deutschland; 2 Institut für Ethik, Geschichte und Theorie der Medizin, Universitätsklinikum Münster, Von-Esmarch-Straße 62, 48149 Münster, Deutschland; 3 Ambulanz für ALS und andere Motoneuronerkrankungen, Charité – Universitätsmedizin Berlin, Augustenburger Platz 1, 13353 Berlin, Deutschland; 4 Institut für Biometrie und Klinische Forschung, Universitätsklinikum Münster, Schmeddingstr. 56, 48149 Münster, Deutschland; 5 Ambulanz für Amyotrophe Lateralsklerose (ALS) und andere Motoneuronerkrankungen, Alfried Krupp Krankenhaus Rüttenscheid, Alfried-Krupp-Strasse 21, 45131 Essen, Deutschland; 6 Ambulanz für Amyotrophe Lateralsklerose u.a. Motoneuronerkrankungen, Universitätsklinikum Münster, Albert-Schweitzer-Campus 1, 48149 Münster, Deutschland; 7 Marienhospital Steinfurt, Klinik für Innere Medizin, Bereich Neurologie, Universitätsklinikum Münster, Mauritiusstrasse 5, 48565 Steinfurt, Deutschland; 8 Klinik für Neurologie, Universitätsmedizin Göttingen, Robert-Koch-Str. 40, 37075 Göttingen, Deutschland; 9 DKD Helios Klinik Wiesbaden, Aukammallee 33, 65191 Wiesbaden, Deutschland; 10 Klinik Hoher Meissner, Hardtstraße 36, 37242 Bad Sooden-Allendorf, Deutschland; 11 Neurozentrum , Lindenhof, Am Lindenhof 2, 53757 Sankt Augustin, Deutschland

**Keywords:** Patientenverfügungen, Suiziderwägungen, Suizidhilfe, Psychosiziale Betreuung, Unterfinanzierung, Advance directives, Suicidal thoughts, Assisted suicide, Psychosocial support, Underfunding

## Abstract

**Hintergrund:**

Die amyotrophe Lateralsklerose (ALS) ist eine Erkrankung, die weiterhin vorwiegend symptomatisch bzw. palliativ behandelt werden muss. Umso wichtiger ist es, neben der Initiierung von Therapien wie Perkutane endoskopische Gastrostomie (PEG), nichtinvasiver Beatmungstherapie (NIBT), invasiver Beatmungstherapie mit Tracheotomie (IBT), beizeiten über die mögliche Beendigung dieser Maßnahmen zu sprechen.

**Fragestellung:**

Welche Bedeutung haben Patientenverfügungen für die Betroffenen und wo liegen eventuelle Defizite der Therapieplanung für das Lebensende?

**Material und Methode:**

An sechs Behandlungszentren wurden im Zeitraum zwischen März 2017 und Januar 2019 Patienten mit der klinisch sicheren Diagnose ALS gebeten, einen Fragebogen auszufüllen. Insgesamt haben 328 Personen diese Bögen ausgefüllt zurückgegeben.

**Ergebnisse:**

Insgesamt 72 % der Befragten besaßen eine Patientenverfügung (PV), 25 % planten eine solche auszufüllen, lediglich 3 % lehnten dies ab. Beim Verfassen der PV hatten die meisten Patienten (90 %) Unterstützung erhalten, bei 56 % fand jedoch keine ärztliche Beratung statt und lediglich 18 % hatten ihre Verfügung gemeinsam mit Arzt und Angehörigen erstellt, wobei sich die Mehrzahl eine Unterstützung auch durch einen Arzt gewünscht hätte. 37 % aller Patienten wünschten sich einen Ansprechpartner, um über ihre Erkrankung zu sprechen, lediglich 40 % von diesen hatten einen solchen Ansprechpartner. 22 % aller Patienten gaben an, einen Suizid erwogen zu haben. Von diesen gaben 55 % an, keinen Ansprechpartner für die psychische Belastung durch die Erkrankung zu haben; 31 % gaben aber an, dass sie gerne einen Ansprechpartner hätten.

**Diskussion und Schlussfolgerung:**

Eine koordinierte Versorgung von ALS-Patienten, die auch die psychosozialen Aspekte in den Blick nimmt, ist dringend erforderlich.

## Abstract (en)

**Background:**

Amyotrophic lateral sclerosis (ALS) is a disease that still has to be primarily treated symptomatically or palliatively. It is therefore all the more important, in addition to initiating treatment, such as percutaneous endoscopic gastrostomy (PEG), noninvasive ventilation therapy (NIVT) and invasive ventilation therapy via tracheotomy (IVT), to discuss the possible termination of these measures early on.

**Question:**

What is the importance of advance directives for those affected and where are possible deficits in therapy planning for the end of life?

**Material and method:**

Between March 2017 and January 2019 patients with a clinically confirmed diagnosis of ALS at six treatment centers were asked to fill out a questionnaire. A total of 328 people returned the completed forms.

**Results:**

Of the participants 72% had already made an advance directive (AD), 25% planned to fill one out and only 3% refused to do so. In composing the AD most patients (90%) had support, although 56% lacked medical counselling and only 18% had drawn up the will together with the doctor and relatives, with the majority of the rest also wanting support from a doctor. A total of 37% of all patients wanted a contact person to talk about their illness but only 40% of them had such a contact person. Of the patients 22% stated that they had considered suicide and of these only 55% stated that they had no contact person for the psychological stress caused by the illness but 31% wished to have such a person.

**Discussion and conclusion:**

A coordinated care of ALS patients, which also takes the psychosocial aspects into account is urgently needed.

Die komplexe Behandlung von Patienten mit einer amyotrophen Lateralsklerose (ALS) bedarf der hausärztlichen, fachärztlichen und spezialärztlichen Zusammenarbeit sowie der multiprofessionellen Versorgung in spezialisierten ALS-Zentren [4]. Eine wichtige Aufgabe besteht darin, ALS-Patienten in ihren persönlichen Entscheidungen über das Beginnen, Begrenzen oder Beenden lebensverlängernder Maßnahmen zu beraten und zu unterstützen.

## Methodik

### Studiendesign

Die Untersuchung wurde als prospektive multizentrische Beobachtungsstudie durchgeführt. In die Studie wurden Patienten mit behandlungsseitig klinisch gesicherter Diagnose ALS (ICD-10 G12.2) aufgenommen. An sechs Behandlungszentren (fünf ALS-Ambulanzen und eine Rehabilitationsklinik für neuromuskuläre Erkrankungen) wurden ALS-Patienten im Erhebungszeitraum zwischen März 2017 und Januar 2019 in die Beobachtungsstudie aufgenommen. Nach Studieninformation und Einwilligung wurden Fragebögen ausgehändigt und ggf. nach dem Ausfüllen zurückgegeben. Wie viele Bögen mithilfe von Angehörigen oder aber gar nicht ausgefüllt wurden, ist nicht dokumentiert. Ebenso ist nicht dokumentiert, wie viele Bögen ausgehändigt, aber nicht zurückgegeben worden sind. Alle eingeschlossenen Bögen wurden pseudonymisiert.

### Untersuchte Variablen

#### Vorhandensein einer Patientenverfügung

Erfasst wurden Fehlen, Ablehnung oder Besitz (samt Dauer) einer Patientenverfügung (PV) sowie deren subjektiv beurteilte Vollständigkeit.

#### Unterstützung beim Erstellen einer PV

Erfasst wurde, ob Patienten hierbei ärztliche, notarielle oder anderweitige Hilfe erfahren haben und was sie sich hierfür wünschen würden (Auswahloptionen mit Möglichkeit der Mehrfachnennung). Weiterhin wurde der Hinterlegungsort der PV erfragt.

#### Einstellung zu lebensverlängernden Maßnahmen

Für Ernährung über eine perkutane endoskopische Gastrostomie (PEG), eine nichtinvasive Beatmungstherapie (NIBT) sowie eine invasive Beatmungstherapie über Tracheotomie (IBT) wurde systematisch erfragt, ob sie gewünscht würden. Dabei wurde zwischen grundsätzlicher Ablehnung und kontextbezogenem Einsatz/Abbruch unterschieden. Zusätzlich wurden konkrete „Umstände“ erfragt, unter denen die Maßnahmen beendet werden sollten (Mehrfachantworten).

#### Suizidüberlegungen

„Erwägungen“ eines möglichen Suizids wurden mit einer Auswahloption – ohne weitere zeitliche und inhaltliche Spezifizierung – erfasst. Weiterhin wurde der Wunsch nach Zulässigkeit von Suizidhilfe durch den eigenen Arzt erfragt.

#### Ansprechpartner bei psychischer Belastung

Erfragt wurde der Wunsch nach einem „Ansprechpartner“, um über „psychische Belastungen“ sprechen zu können. In einer Folgefrage wurde ermittelt, ob der gewünschte Ansprechpartner vorhanden sei.

#### Demographische und klinische Charakteristika

Die erhobenen Charakteristika der untersuchten Patienten sind in Tab. 1 zusammengefasst.

**Tab. 1:** Demographische und klinische Daten

**Alter**	< 50 Jahre: 10 % (*N* = 30)	50–70 Jahre: 58 % (*N* = 173)	> 70 Jahre: 32 % (*N* = 97)	Gesamtzahl = 300 KA (keine Angabe) = 28
**Geschlecht**	Männlich 61,5 % (*N* = 184)	Weiblich 38,5 % (*N* = 115)	–	Gesamtzahl = 299 KA = 29
**Zeit seit Diagnosestellung**	< 1 Jahr: 29 % (*N* = 96)	1–3 Jahre: 32 % (*N* = 105)	> 3 Jahre: 30 % (*N* = 99)	Gesamtzahl = 300 KA = 28
**Region der Betroffenheit **(mit Mehrfachnennungen)	Bulbäre Symptome: 49 % (*N* = 147)	Extremitätenparesen Arme: 64 % (*N* = 190) Beine: 66 % (*N* = 198)	Respiratorische Symptome 22 % (*N* = 67)	Gesamtzahl = 298 KA = 30
**Bereits eingeleitete lebensverlängernde Maßnahmen**	Nichtinvasive Beatmung 16 % (*N* = 52)	Invasive Beatmung 3 % (*N* = 9)	PEG-Ernährung: 11 % (*N* = 37)	Gesamtzahl = 300 KA = 28

### Statistische Analysemethoden

Die pseudonymisierten Daten wurden strukturiert erfasst und mit SPSS (SAS 9.4/SAS Institute, SPSS 27/IBM, Armonk, NY, USA) ausgewertet.

## Ergebnisse

### Vorhandensein einer Patientenverfügung

Zum Zeitpunkt der Befragung besaßen 72 % (*n* = 235) eine PV. Weitere 25 % (*n* = 83) beabsichtigten eine PV zu erstellen, während eine kleine Patientengruppe (3 %, *n* = 9) dies ablehnte. Ein Zusammenhang mit soziodemographischen Merkmalen oder mit der Zeitspanne seit Diagnosestellung war nicht nachweisbar. Bemerkenswert war der Zusammenhang zum Grad der Betroffenheit: 81 % (*n* = 54) der Patienten mit respiratorischer Beteiligung hatten bereits eine PV erstellt. Von den PV-Besitzern hielten 87 % (*n* = 204) ihre PV für (weitgehend) vollständig; 79 % (*n* = 184) von ihnen hatten eine zusätzliche Vorsorgevollmacht.

### Unterstützung beim Erstellen einer PV

**Abb. 1:**
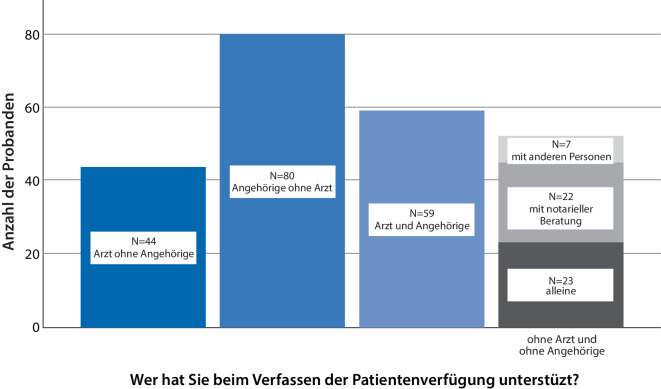
Unterstützung bei der Erstellung einer Patientenverfügung

Die Mehrheit der ALS-Patienten hatte Unterstützung beim Verfassen ihrer PV (90 %, *n* = 212, Abb. 1) – an erster Stelle durch Angehörige, gefolgt von Ärzten. Ganz *ohne* ärztliche Beratung verfassten 56 % (*n* = 132) der Patienten ihre PV. Von dieser Patientengruppe hätten sich 54 % (*n* = 71) ärztliche Unterstützung gewünscht, während 35 % (*n* = 46) ausdrücklich darauf verzichtet hatten. Hinterlegt waren die PV (Mehrfachnennungen erlaubt) mehrheitlich bei den „medizinischen Unterlagen“ (68 %, *n* = 159) oder den Angehörigen (44 %, *n* = 103), deutlich seltener beim Hausarzt (23 %, *n* = 53), beim Vorsorgebevollmächtigten (23 %, *n* = 53), in einem Register (17 %, *n* = 40) oder beim Notar (14 %, *n* = 32).

### Planung lebensverlängernder Maßnahmen

**Abb. 2:**
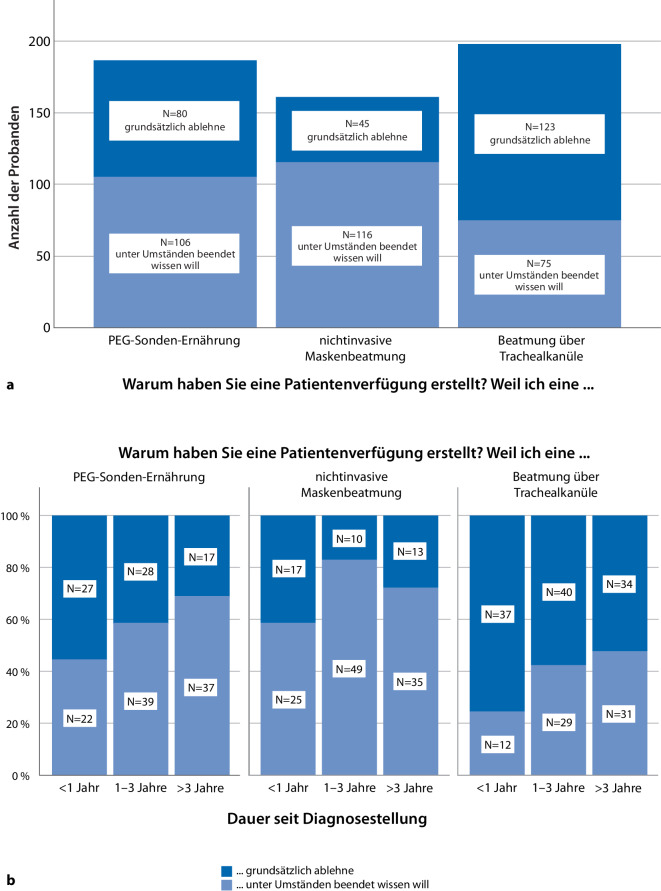
**a** Festlegungen zur Therapiebegrenzung: Häufigkeit in Bezug auf spezifische Therapiemaßnahme vs. grundsätzliche Ablehnung gegenüber Beendigung unter bestimmten Bedingungen. **b** Festlegungen von Therapiebegrenzung im Krankheitsverlauf: Dauer seit Diagnosestellung vs. grundsätzliche Ablehnung gegenüber Beendigung unter bestimmten Bedingungen. *PEG* perkutane endoskopische Gastrostomie

Vorhandene Patientenverfügungen (*n* = 235) wurden häufig genutzt, um Festlegungen über Ernährung durch eine PEG (79 %; *n* = 186) sowie über eine nichtinvasive (69 %; *n* = 161) bzw. invasive Beatmungstherapie (84 %; *n* = 198) zu treffen (Abb. 2a). Dabei wollte ein hoher Anteil an Patienten die Option unterstreichen, zuvor eingeleitete lebensverlängernde Maßnahmen beenden zu können – insbesondere bei der nichtinvasiven Beatmung (49 %, *n* = 116). Für alle drei Maßnahmen wurden teils grundsätzliche Ablehnung und teils der Wunsch nach kontextabhängiger Beendigung geltend gemacht. Hierbei zeigt sich für alle drei Interventionen keine Korrelation mit dem Grad der Betroffenheit, wohl aber mit der Zeitspanne seit Symptombeginn. So nahm die pauschale Opposition gegenüber lebensverlängernden Maßnahmen (insbesondere einer PEG) im Krankheitsverlauf ab, während die Option einer kontextabhängigen Beendigung bereits eingeleiteter Maßnahme häufiger in Betracht gezogen wurde (Abb. 2b). Bei den konkreten „Umständen“, unter denen die lebensverlängernden Maßnahmen beendet werden sollten (Mehrfachnennung), dominierten der „unmittelbare Sterbeprozess“ mit 69 % (*n* = 163), ein vorliegendes „Koma“ mit 65 % (*n* = 152), der „irreversible Verlust der Bewegungsfähigkeit“ mit 46 % (*n* = 109) und ein „irreversibler Kommunikationsverlust“ mit 46 % (*n* = 108).

### Suiziderwägung

**Abb. 3:**
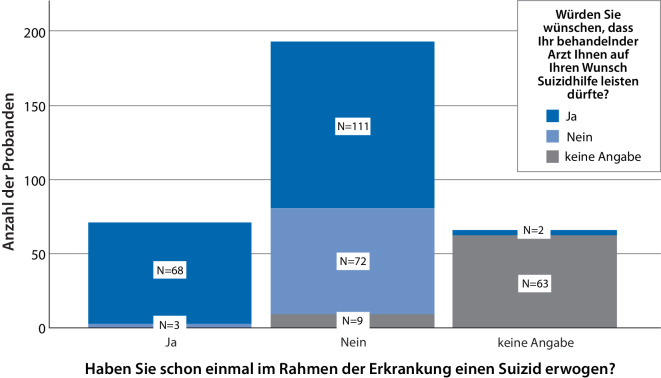
Häufigkeit von Suiziderwägungen vs. Präferenz für mögliche ärztliche Suizidhilfe

Von allen 328 Befragten gaben 59 % (*n* = 192) an, keinen Suizid erwogen zu haben, 22 % (*n* = 71) bestätigten jedoch eine solche Überlegung, 19 % machten keine Angaben (*n* = 65). Eine Korrelation mit der Erkrankungsdauer, dem Grad der Betroffenheit oder dem Besitz einer PV war dabei nicht auszumachen. Wann im Verlauf besagte Suiziderwägungen stattgefunden hatten, wurde nicht erfragt. Des Weiteren wünschten 55 % (*n* = 181) der Gesamtkohorte sowie 96 % (*n* = 68) der Patienten mit Suiziderwägung, dass ihrem Arzt Suizidhilfe erlaubt sein sollte (Abb. 3). Suizidhilfe „in einem anderen Land, z. B. in der Schweiz“ wurde von 28 % (*n* = 92) aller Befragten als eine für sie denkbare Option gesehen sowie von 63 % (*n* = 45) derjenigen Patienten, die einen Suizid bereits in Erwägung gezogen hatten. Patienten, die wünschten, ärztliche Suizidassistenz solle erlaubt sein, würden zu 50 % (*n* = 90) Suizidassistenz auch außerhalb Deutschlands in Betracht ziehen.

### Ansprechpartner bei psychischer Belastung

**Abb. 4:**
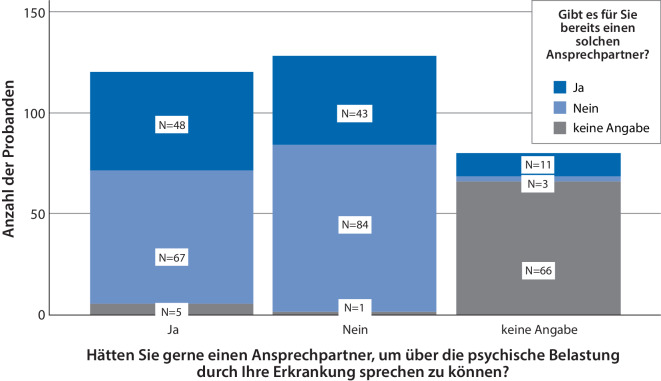
Wunsch nach Ansprechpartner bei psychischen Belastungen vs. Vorhandensein eines Ansprechpartners: Wunsch und Wirklichkeit

Insgesamt 37 % (*n* = 120) der Patienten bekundeten den Wunsch nach einem Ansprechpartner, um über psychische Belastungen im Zusammenhang mit ihrer ALS zu sprechen (24 % [*n* = 80] machten keine Angaben). Nur eine Untergruppe der Patienten mit Wunsch nach einem Ansprechpartner gab an, dass ein entsprechender Ansprechpartner für sie vorhanden sei (40 %, *n* = 48, Abb. 4**)**; 56 % (*n* = 67) gaben an, dass kein solcher Ansprechpartner existiert, das sind von allen 328 befragten Patienten 20 %.

Unter den Patienten, die angaben, einen Suizid „in Erwägung“ gezogen zu haben (*n* = 71), gaben 55 % an, keinen Ansprechpartner für die psychische Belastung durch die Erkrankung zu haben; 31 % gaben aber an (*n* = 22), dass sie gerne einen Ansprechpartner hätten, 24 % dagegen hielten dies nicht für erforderlich. Ein Zusammenhang dieser Antworten mit soziodemographischen Merkmalen, der Erkrankungsdauer, der Betroffenheit oder dem Vorliegen einer PV war nicht auszumachen.

Allerdings zeigte sich bei der Mehrheit eine enge familiäre Einbindung: 72 % (*n* = 236) aller Befragten waren verheiratet oder in einer Partnerschaft. 79 % (*n* = 259) hatten Kinder, zu denen in 90 % (*n* = 233) „enger Kontakt“ bestünde.

### Demographische und klinische Daten

An der Studie nahmen 328 Patienten teil. Die Geschlechter- und Altersverteilung entspricht einer typischen ALS-Kohorte (61,5 % Männer, 38,5 % Frauen; 10 % der Betroffenen jünger als 50 Jahre, 32 % über 70 Jahre, die übrigen zwischen 50 und 70 Jahren). Der Anteil an Betroffenen, der bereits lebensverlängernde Maßnahmen in Anspruch genommen hatte (11 % PEG, 16 % NIBT und 3 % IBT), war geringer als etwa in einer aktuellen Studie der Charité (29 %, 21 % bzw. 10 % [2]). Somit wurden hier auch Patienten in einem vergleichsweise frühen Stadium der ALS erfasst. Die demographischen und klinischen Daten sind in Tab. 1 zusammengefasst.

## Diskussion

Amyotrophe Lateralsklerose ist gegenwärtig nicht heilbar, dennoch bestehen zahlreiche Behandlungsoptionen, vorwiegend zur Symptomlinderung, Verbesserung der sozialen Teilhabe und Verlängerung der Lebenszeit. Ob, wann und wie lange ALS-Patienten solche Maßnahmen, insbesondere PEG- und Beatmungstherapien, in Anspruch nehmen wollen oder nicht, sollte – darüber besteht Einigkeit – von ihren persönlichen Wertvorstellungen abhängen und vor dem Hintergrund guter medizinischer Aufklärungs- und Beratungsgespräche und bestmöglicher psychosozialer Unterstützung ausgelotet werden. Zudem sollte immer wieder Gelegenheit geboten werden, Überlegungen und Präferenzen zum Therapieregime in einer Patientenverfügung (PV) zu dokumentieren und im Verlauf der Erkrankung zu überdenken und zu revidieren – wie dies der Idee der Vorsorgeplanung („advanced care planning“) entspricht [3].

Zur Realität solcher Vorsorgeplanungen bei ALS-Patienten sowie zu Suiziderwägungen oder dem Wunsch nach ärztlicher Suizidhilfe gibt es bisher wenige Untersuchungen [1]. In der vorliegenden systematischen Erhebung wird ein Teil dieser Themen adressiert.

Die Ergebnisse dieser Studie wurden durch strukturierte Interviews mit schriftlichen Fragen und vordefinierten Antwortoptionen erzielt. Durch dieses Studiendesign konnte eine umfangreiche Kohorte im Rahmen ihrer Regelversorgung an spezialisierten Behandlungszentren befragt werden. Diese Untersuchungsmethodik ist zugleich mit Limitationen verbunden: Fragestellungen wie Antwortmöglichkeiten beruhten auf empirischen Erfahrungen der Autoren; qualitative Daten konnten nur zum Teil gewonnen werden; die selektive Rekrutierung an spezialisierten Zentren bringt mögliche Verzerrungen mit sich.

Zum Zeitpunkt der Befragung besaßen 72 % (*n* = 235) der Patienten eine PV. Dies stimmt mit Erhebungen aus den USA und Kanada überein, in denen 70–90 % der ALS-Patienten über eine PV verfügten [4–7]. Für Deutschland und Europa gibt es nur wenige Untersuchungen, diese erbrachten eine deutlich geringere PV-Häufigkeit [8–11]. Die hohe Erstellungsrate in unserer Studie zeigt die grundsätzliche Bedeutung einer PV bei ALS. Sie wurde hier häufig und bereits zu Beginn der Krankheit erstellt, obwohl deren Verlauf den betroffenen Patienten in der Regel die Möglichkeit belässt, Behandlungsentscheidungen – teilweise mit Unterstützung durch elektronische Kommunikationssysteme – jeweils aktuell und selbstbestimmt zu treffen. Daher kommen PV wohl selten zur unmittelbaren Anwendung: etwa bei akuten Beatmungsnotfällen oder bei plötzlich hinzukommenden Erkrankungen, die zum Verlust der Einwilligungsfähigkeit führen. Die Bedeutung von PV liegt somit meist weniger in ihrer Funktion zur Regulierung von Therapiemaßnahmen als in ihrer Funktion, Entscheidungsprozesse im Sinne des „advanced care planning“ [3] anzustoßen und einen Rahmen für die palliativmedizinische Betreuung zu bahnen [12, 13]. Idealerweise dient dann die PV der Grundidee gemeinsamer Entscheidungsfindung von Arzt und Patient und wird zum Katalysator für kontinuierliche Überlegungen, Gespräche und Festlegungen.

Insgesamt 87 % der Patienten betrachteten ihre PV als inhaltlich (weitgehend) vollständig. Dieses Ergebnis ist wenig erstaunlich, da von den rekrutierenden ALS-Behandlungszentren (und auf deren Internetseiten) eine Muster-PV bereitgestellt wird, in der typische Entscheidungssituationen zur invasiven Ernährung und Beatmung präzisiert und unterschieden werden [1, 14]. Die Nutzung einer PV zur Dokumentation von Wünschen nach interventions- und kontextspezifischen Therapiebegrenzungen zeigt eine erhebliche Präferenzvariabilität: So wurden die erfragten drei Interventionen in unterschiedlichen Anteilen primär abgelehnt (34 % PEG, 19 % NIBT, 52 % IBT) oder sollten unter bestimmten Bedingungen beendet werden (45 % PEG, 49 % NIBT, 32 % IBT).

Ein kritisches Ergebnis unserer Untersuchung besteht darin, dass lediglich 25 % (*n* = 59) der Patienten ihre PV in gemeinsamer Entscheidungsfindung mit Arzt und Angehörigen erstellt hatten. Ganz ohne ärztliche Unterstützung haben 56 % der ALS-Patienten ihre PV verfasst, von denen mehr als die Hälfte diese Tatsache ausdrücklich bedauerte. Dass somit etwa ein Drittel der befragten ALS-Patienten auf eigentlich erwünschte ärztliche Unterstützung verzichten musste, werten wir als ernstes Defizit. Dieses Ergebnis weist auf die begrenzten zeitlichen und personellen Ressourcen und die Unterfinanzierung in der ambulanten Versorgung von Patienten mit ALS hin. Auch die anzustrebende Einbeziehung der Hausärzte in die Erstellung und Dokumentation einer PV ist nur bedingt gelungen: Lediglich ein Fünftel der Befragten hatten ihre PV (auch) dem Hausarzt übergeben. Etwa ein Drittel der Patienten hatten ihre PV beim Notar oder in einem Register hinterlegt. Beide Dokumentationsorte versprechen hohe Rechtssicherheit, sind im akuten Notfall jedoch schlecht erreichbar.

Insgesamt 22 % der von uns befragten ALS-Patienten bejahten, „im Rahmen ihrer Erkrankung schon einmal einen Suizid erwogen“ zu haben. Dabei lässt der Begriff „Erwägung“ allerdings offen, wie konkret und persistent diese Überlegungen waren. Zudem befanden 55 % der ALS-Patienten, Suizidhilfe durch ihren Arzt solle rechtlich erlaubt sein. Allerdings lässt das Studiendesign keinen Aufschluss darüber zu, in welchem Maße diese Präferenz eine ethische Wertvorstellung oder einen konkreten Wunsch nach potenzieller Suizidhilfe zum Ausdruck bringt. Die vorliegende Erhebung fiel in jenen Zeitraum von 2015 bis 2020, in dem in Deutschland „geschäftsmäßige Suizidhilfe“ (und damit auch eine nichtgewinnorientierte, aber auf grundsätzlicher Bereitschaft basierende Suizidassistenz durch Ärzte) vorübergehend strafrechtlich untersagt war [29]. In diesem Zusammenhang ist die Frage nach Inanspruchnahme von Suizidhilfe „in der Schweiz“ zu sehen, die sich 28 % der Befragten vorstellen konnten. Durch das Urteil des Bundesverfassungsgerichts von 2020 (BVerfG., Rz.204ff., [30]) hat sich eine veränderte Situation ergeben, die sich im Befragungsdesign und -ergebnis naturgemäß noch nicht wiederfindet.

Daten anderer Studien zur Erwägung eines Suizids durch ALS-Patienten zeigen allgemeine Suiziderwägungen bei 39–50 % und konkrete Suizidgedanken bei 6–26 % der Betroffenen [17–19]. Untersuchungen aus den USA (Oregon und Washington) berichteten von Suiziderwägung bei 56 % der erfassten ALS-Patienten und von realisierten assistierten Suiziden bei 5 % [5, 20]. In den Niederlanden wird die Häufigkeit von ärztlich unterstütztem Suizid oder aktiver Sterbehilfe (beides dort legal) bei ALS mit 16,8 % angegeben [21]. Für Dänemark und Schweden (ärztliche Suizidhilfe verboten) wurde eine – gegenüber der Gesamtbevölkerung – 5‑fache Erhöhung der Suizidrate ermittelt [22–24]. In Deutschland wurde bisher keine erhöhte Suizidhäufigkeit nachgewiesen, wobei nur wenige Daten vorliegen [25]. Weitere Untersuchungen sind notwendig, um den möglichen Zusammenhang von Suiziderwägung (und Suizid) mit einem depressiven Syndrom zu klären. Bisherige empirische Studien konnten jedoch zeigen, dass Suiziderwägungen mehrheitlich nicht auf dem Boden einer klinischen Depression entstehen [26]. Wichtig ist es zudem, die Gründe für Suizidwünsche von ALS-Patienten zu verstehen [27, 28], nicht zuletzt um ihnen angemessene Hilfsangebote machen zu können.

Insgesamt weisen die Ergebnisse dieser Studie auf erhebliche Defizite im Bereich der „sprechenden“ und „zuhörenden“ Medizin hin, obwohl eine koordinierte Versorgung von ALS-Patienten nicht nur ihre Lebensqualität, sondern auch ihr Überleben statistisch signifikant (um 7,5 bzw. 10 Monate) erhöht [15, 16]. Komplexe Beratungs- und kooperative Planungsleistungen auch durch nichtärztliche Akteure (ALS-Nurses) sind in der bisherigen Vergütungssystematik nicht oder nur unzureichend abgebildet. Als Hinweis auf die defizitären Ressourcen ist auch das Fehlen von Ansprechpartnern für „die psychische Belastung durch die Erkrankung“ bei 20 % aller befragten ALS-Patienten (und bei 31 % aller Patienten mit Suiziderwägung) zu sehen: Die Vermittlung in die ambulante psychotherapeutische Betreuung ist aufwendig und mit langen Wartezeiten verbunden.

## Fazit für die Praxis

- Beinahe alle der von uns befragten ALS-Patienten hatten eine Patientenverfügung bzw. planten, eine solche zu erstellen.
- ALS-Patienten wünschen, frühzeitig eine Patientenverfügung zu erstellen.
- Das Aufsetzen einer PV sollte gemeinsam – bevorzugt mit dem behandelnden ALS-Spezialisten und den Angehörigen bzw. Bevollmächtigten – geschehen.
- Das frühzeitige und gemeinsame Erstellen einer PV dient neben der Klärung des Vorgehens im Notfall vor allem dem gemeinsamen Gespräch über das von den Patienten gewünschte weitere Therapieregime („advanced care planning“).
- Die PV sollte im Krankheitsverlauf immer wieder besprochen und aktualisiert werden.
- Eine ALS-spezifische PV ist zu bevorzugen, um die ALS-relevanten Entscheidungen zu dokumentieren (z. B. https://als-charite.de/wp-content/uploads/2020/03/ALS-Ambulanz_Patientenverfuegung_V3_1_druck_02_FINAL.pdf).
- Suiziderwägungen sind bei nicht wenigen ALS-Patienten vorhanden, häufig auch die Erwägung der Inanspruchnahme im Ausland. Die Mehrheit der Befragten wünscht, dass die ärztliche Suizidhilfe in Deutschland erlaubt sein sollte.
- Die psychosoziale Betreuung der ALS-Patienten ist unzureichend und dringend verbesserungsbedürftig. Hierfür muss ein institutioneller Rahmen für eine integrierte Versorgung mit ausreichender Vergütung geschaffen werden (analog der Psycho-Onkologie bei Tumorpatienten).

## Data Availability

Die erhobenen Datensätze können auf begründete Anfrage in anonymisierter Form beim korrespondierenden Autor angefordert werden. Die Daten befinden sich auf einem Datenspeicher am Institut für Biometrie und Klinische Forschung (IBKF) der Universität Münster, Schmeddingstraße 56 in 48149 Münster.
